# Effect of home‐based exercise with or without a Mediterranean‐style diet on adiposity markers in postmenopausal women: A randomized‐control trial

**DOI:** 10.14814/phy2.70239

**Published:** 2025-02-07

**Authors:** Abbigail Tan, Gareth Dunseath, Rebecca L. Thomas, Sarah L. Prior, Richard M. Bracken, Rachel Churm

**Affiliations:** ^1^ Applied Sports Technology, Exercise and Medicine (A‐STEM) Research Centre, Faculty of Science and Engineering Swansea University Swansea UK; ^2^ Diabetes Research Group, Grove Building Swansea University Swansea UK

**Keywords:** abdominal obesity, adipokines, adipose tissue, cardiometabolic disease, cardiometabolic health, lifestyle intervention, postmenopausal women, women's health

## Abstract

Advancing age and estrogen deficiency increases susceptibility of post‐menopausal women (PMW) to abdominal obesity and manifestation of cardiometabolic disease. There is limited evidence on the effect of lifestyle interventions on adiposity markers within at‐risk PMW. Therefore, this study aims to evaluates an 8‐weeks of home‐based, equipment‐free, interval training (HEFIT) with or without Mediterranean‐style diet (MD) on adiposity markers in physically inactive, postmenopausal women with overweight/obesity. Thirty PMW (56.7 ± 3.9 years, BMI: 30.5 ± 5.2 kg/m^2^) were randomly assigned to three groups: (i) Ex; HEFIT thrice weekly/week, (ii) EX + MD, or (iii) CTL; control. Visceral Adiposity Index (VAI), body weight, BMI, waist and hip circumference (WC; HC), visceral adipose tissue (VAT), total body fat percentage, leptin, and adiponectin were determined pre‐ and post‐8‐week intervention. There was no significant between group effect on VAI. Compared to CTL, a significant between group reduction was seen in weight, BMI, and WC in both EX and EX+D (*p* < 0.05). Leptin and adiponectin remained unchanged in all groups (*p* > 0.05). Adherence rates were 85% and 96% for EX and EX+MD, respectively, and 80% of EX+D of participants had optimal adherence to diet. Concluding HEFIT with or without dietary changes could improve adiposity in overweight/obese postmenopausal women.

## 1 INTRODUCTION

With a median age of menopause onset between 45 and 55 years (Faubion et al., 2015) and the average life expectancy increasing, there is an ever‐increasing amount of time women will spend in a post‐menopausal state. Consequently, this could have a significant impact on an individual's physical and mental health. Hormonal fluctuations associated with menopause transition, age, and environmental factors are associated with increased risk of obesity and increased adiposity. There is substantial evidence that these physiological and metabolic alteration are the result of altered energy consumption, reduced fat oxidation rates, and detrimental adipose tissue redistribution (Opoku et al., 2023).

Adipose tissue is an important endocrine and essential organ for energy homeostasis regulation. Subcutaneous adipose tissue (SAT) is the largest and preferential depot for excess fat and long‐term lipid storage, while visceral adipose tissue (VAT) is highly metabolically active and acts as an acute‐response supplier of free fatty acids (Chait & den Hartigh, 2020). The distribution of adipose tissue in the human body play an important role and indicator in metabolic profile. SAT distribution localized primarily to the upper and lower body region is associated with a better metabolic profile and higher insulin sensitivity (Laforest et al., 2015), while VAT distribution localized within the abdominal region is associated with metabolic dysfunction and insulin resistance (IR) (Ledoux et al., 2010).

Adipose tissue dysfunction is associated with numerous deleterious effects that contributes to metabolic dysfunction. Hypertrophic VAT secretes pro‐inflammatory adipokines like leptin while reducing anti‐inflammatory adipokines secretion like adiponectin, leading to local and systemic inflammation and further metabolic disturbances (Lipke et al., 2022). Leptin, proportional to fat mass, regulates energy balance and glucose‐insulin metabolism via the hypothalamus. In obesity, hyperleptinaemia leads to leptin resistance, causing excess triglycerides to accumulate in non‐adipose and adipose tissue, subsequently impairing insulin sensitivity and secretion (Steinberg et al., 2002). Conversely, adiponectin is inversely proportional to total fat mass and possesses cardioprotective effects including improving muscle and hepatic insulin sensitivity (Straub & Scherer, 2019).

Abdominal obesity is associated with metabolic syndrome (MetS) and is driven by factors including IR, dyslipidaemia, inflammation, and adverse cardiovascular effects that contribute to the manifestation of cardiometabolic disease (CMD) (Valenzuela et al., 2023). Due to the loss of protective effects of estrogen and advancing age, post‐menopausal women are at an increased risk of CMD.

Our previous meta‐analysis demonstrated that short‐term exercise interventions as brief as 8 weeks can significantly reduce waist circumference (WC), a MetS indicator, in post‐menopausal women (Tan et al., 2023). Similarly, dietary interventions alone have also shown to improve central adiposity, as evidenced by reductions in WC and VAT in this high‐risk population (Amiri et al., 2022). Generally, long‐term lifestyle behavioral programs combining both diet and exercise are superior in benefiting those with abdominal obesity (Johns et al., 2014), yielding better overall cardiometabolic profiles than diet or exercise alone (Cheng et al., 2018; Khalafi & Symonds, 2023).

The 2020 European Menopause and Andropause Society (EMAS) position statement summarized the existing evidence on the Mediterranean diet (MD) and menopausal‐related health. Thus, highlighting the associations between short‐term and long‐term adherence with a reduced cardiovascular risk and all‐cause mortality in both menopausal and post‐menopausal women (Lipke et al., 2022). In support, an umbrella review of meta‐analyses reported the superior effectiveness of MD in improving WC compared to controlled diets (Dinu et al., 2018). Additionally, high‐intensity interval training (HIIT) has been reported to be superior to traditional moderate‐intensity training (MIT) in reducing abdominal adiposity (Straub & Scherer, 2019; Valenzuela et al., 2023), making it a time‐efficient modality against cardiometabolic risk. The synergistic effects of incorporating both lifestyle behaviors may offer targeted strategies to prevent and benefit cardiometabolic health outcomes. Yet, research is limited in the evaluation of the combined effects of potentially low‐cost, home‐based high‐intensity interval training options, and the Mediterranean diet (MD) in postmenopausal women.

Therefore, this study aimed to investigate the effects of adiposity markers with an 8‐week, home‐based, equipment‐free IT (HEFIT) and MD in physically inactive, post‐menopausal women with overweight or obesity. In this randomized‐controlled trial, we hypothesized that (1) combined HEFIT and MD (EX+D) will improve adiposity markers and (2) EX+D and EX will result in greater magnitude in improvements in adiposity markers compared to CTL. We hypothesize that (1) EX+D will improve adiposity markers and (2) EX+D and EX will result in greater magnitude in improvements in adiposity markers compared to CTL.

## 2 MATERIALS AND METHODS

### 2.1 Ethical approval and recruitment

This study was reviewed and received favorable ethical opinion by the United Kingdom's Health Research Authority (Hampstead Research Ethics Committee; 22/LO/0301; IRAS ID: 314505). This study was conducted in accordance with the Declaration of Helsinki and preregistered with ClinicalTrials.gov (NCT05417698). Participant recruitment methods varied and included advertisements via staff email, intranet, social media, and posters. All participants gave written and informed consent prior to participation.

### 2.2 Study design and randomization

This was a three‐arm randomized‐controlled study investigating the cardiometabolic risk markers in an 8‐week HEFIT exercise intervention with/without MD and free‐living control. Conducted by the principal investigator (PI) before study initiation, participants who self‐reported to meet eligibility criteria were randomly assigned by computerized random assignment (randomizer.org) to one of three groups at an allocation of 1:1:1: exercise and MD (EX+D) consisting of exercise for 20 min, three times a week, plus MD; exercise only (EX) consisting of exercise for 20 min, three times a week; or a standard care control group (CTL) who were instructed to maintain their usual level of activity and diet. The PI was not involved in the rest of the trial. Following eligibility determination at the screening visit, the participant's assigned group was revealed. The researcher (A.T.), who conducted all screening and laboratory visits, was blinded to each participant's group allocation until this point to maintain unbiased assessments during initial participant interactions, due to data collection across intervention participant allocations were not blinded to researcher at final visit.

All participants were instructed to maintain their usual caloric intake. To monitor dietary intake without prompting behavioral changes, a 1‐week pre‐study period was including, during which all participants recorded daily food intake using a commercially available app (MyFitnessPal). This allowed participants to normalize tracking before the 8‐week intervention phase. Dietary intake remained consistent across the study period (Table S1), with participants performing baseline assessments in week 1 with repeat assessment at follow‐up at week 8.

### 2.3 Participants

A total of 126 potential participants expressed interest and were sent a participant information sheet (PIS) denoting the full description of the study. The PIS also included the inclusion criteria: post‐menopausal (defined by cessation of menstruation for at least 12 consecutive months) (Davis et al., 2015), aged 45–65 years, BMI >25 kg/m^2^, no known diseases, generally well to exercise, and physically inactive (defined by International Physical Activity Questionnaire (IPAQ) score category I and not engaged in at least 60 min/week of structured exercise in previous 6 months). The main exclusion criteria were as follows: (1) a previous diagnosis of a chronic health disease such as cancer, cardiovascular, kidney, liver, gastrointestinal, diabetes (type 1 or 2), (2) on HRT for <6 months, (3) prescribed medications related to cardiometabolic health conditions including statins, metformin, anti‐hypertensives, or beta‐blocker medications.

**FIGURE 1:**
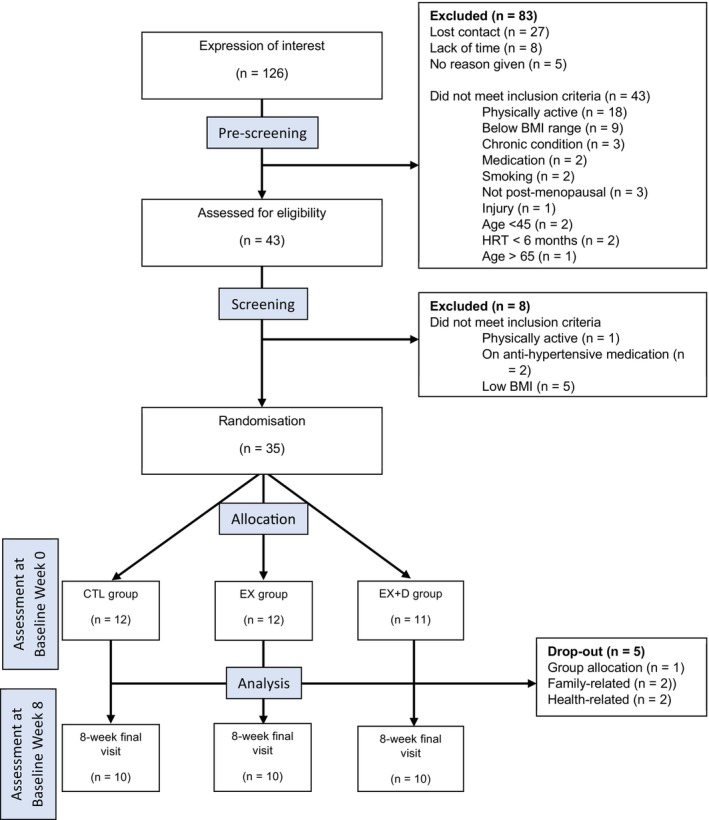
CONSORT flow diagram of participant recruitment and group allocation.

During pre‐screening, 83 potential participants were excluded; did not meet the inclusion criteria (*n* = 43), declined/lost contact (*n* = 32), unable to commit to the study due to lack of time (*n* = 8). In total, 43 potential participants were then invited on study site for screening. Upon screening, self‐reported variables, that is, menopause status, IPAQ, and medical questionnaire, were collected, and height and weight were determined to calculate BMI, all data was screened and verified by the researcher. 8 participants did not meet inclusion criteria: low BMI (*n* = 5), on blood pressure tablet (*n* = 2), physically active (*n* = 1). A total of 35 participants passed screening and were subsequently randomized and allocated to their group assignment. A CONSORT flow diagram depicting recruitment of study participants is denoted in Figure 1.

### 2.4 Home equipment free IT (HEFIT) Programme

The 8‐week HEFIT program required participants to perform unsupervised HEFIT three times/week (total commitment: 24 sessions) with remote digital safety reporting and support from study team. Each session involved a self‐chosen protocol, based on HEFIT, performed at a high intensity training intensity and self‐monitored at ≥80% of maximum heart rate and corresponding to ≥17 Borg rated perceived‐exertion (RPE) scale (Borg, 1982). To accommodate individual preferences and promote adherence, participants had the flexibility to choose between two options:(1) a progressive protocol with the decreasing low‐intensity intervals from Week 1 to 4, maintained from weeks 5 to 8, aiming to complete as many repetitions as possible during the 60 s high intensity (HIIT) intervals, or (2) any HEFIT protocol available on YouTube. YouTube videos were required to meet specific criteria: (1) have “HIIT” in the title of the video; (2) performed without the use of equipment; (3) for video durations that last shorter or longer than 20 min, participants should ensure to repeat the video until 20 min have been completed or pause at the end of an exercise at the end of 20 minutes duration. Participants' adherence to the intervention were recorded with a Garmin Forerunner 35 (Garmin Ltd., United States), which provided data on heart rate and duration of exercise. Adherence and fidelity of the intervention was monitored via average age‐related heart rate with the focus on maintaining the target heart rate range of ≥80% of maximum heart rate during the high‐intensity intervals, rather than solely recording the total exercise duration. This flexible approach was developed in consultation with the public and patients to ensure it was practical and achievable for participants. A comprehensive briefing by the researcher was delivered to all participants prior to the intervention involving familiarization of the exercise types and modality, target heart rates, and RPE. Adherence and fidelity were ensured through self‐reports via an encrypted messenger service (WhatsApp) and fortnightly meetings with the study researcher.

### 2.5 Mediterranean‐style diet and measurement of adherence

In addition to the tri‐weekly exercises, participants in EX+D were instructed to adhere to MD based on the guidance of a specific, short 14‐item questionnaire, the MD adherence (MEDAS) score, used by the Mediterranean Diet Prevention Group (PREDIMED) (Martínez‐González et al., 2002) (Table S2). Specific guidance was based on informing participants of incorporating a diet of meals mainly based around an abundance of plant‐based foods, legumes, and whole grains, moderate consumption of cheese, yoghurt, fish, poultry, and low consumption of red/processed meat and alcohol with a focus of utilizing olive oil as the main source of fat.

To determine the degree of adherence and changes to MD in the EX+D group, participants completed the MEDAS score at the end of each week (from baseline to week 8). This questionnaire scored each MD component to assess individual adherence levels. A value of ‘1’ and ‘0’ were assigned to each item if they were achieved or not achieved, respectively. From the sum of values, the degree of adherence was determined. Optimal adherence to MD was considered high for score ≥9, and low for scores <9 (León‐Muñoz et al., 2012). The full MD adherence score was adjusted to accommodate participants who were vegetarian (*n* = 2) or non‐drinkers (*n* = 2), removing inappropriate items resulted in achieving ≥65% of the total adjusted score was defined as high adherence. This percentage‐based adjustment allowed us to fairly assess adherence levels across participants with different dietary restrictions.

### 2.6 Outcome measures

All outcome measures were assessed at baseline and after the 8‐week intervention. Participants reported to the laboratory following a 12‐h overnight fast (from 20:00 h) and having abstained from alcohol 24 h before for all visits.

Visceral adiposity index (VAI) was the primary outcome of the trial and calculated with the following equation:

$$\frac{\text{Waist circumference}\;\left(\mathrm{cm}\right)}{36.58+\left(1.89\times \mathrm{BMI}\right)}\times \frac{\mathrm{TG}\;\left(\text{mmol}/L\right)}{0.81}\times \frac{1.52}{\mathrm{HDL}\;\left(\text{mmol}/L\right)}$$

#### 2.6.1 Body composition

Body weight, height, waist and hip circumference, total body fat percentage, lean tissue mass, and VAT were assessed. Participant's waist circumference (WC) and hip circumference (HC) were measured according to World Health Organization (WHO) guidelines, measured at the approximate midpoint between the iliac crest and the last rib, and at the maximum circumference of the buttocks, respectively. The dual energy x‐ray absorptiometry (DEXA) (Stratos dR, Diagnostic Medical Systems (DMS) Group, France) was used to measure total body fat percentage, lean tissue mass, and VAT.

#### 2.6.2 Leptin and adiponectin

Venous fasting blood sample collected to yield plasma (EDTA K2 BD Vacutainer®). Fasting whole blood samples were immediately placed on ice following blood draw and processed within 15 min of collection. Blood tubes were centrifuged at 5000 rpm for 5 min at 4°C to yield plasma. Fasting leptin and adiponectin levels were determined using commercial human‐specific DuoSet ELISA kits (R&D Systems: Cat# DY1065 and Cat# DY398, respectively), with an intra‐assay coefficient of variation of 9.8% for leptin and 1.4% for adiponectin.

### 2.7 Statistical analysis

A prior power analysis was conducted in GPower 3.1 to determine the required sample size for detecting a statistically significant effect in the context of a three‐arm randomized controlled trial (RCT) using repeated measure analysis of variance (ANOVA). Based on previous unpublished feasibility data (*n* = 10), which assessed HEFIIT over an 8‐week period in PMW, the expected effect size for change in waist circumference was estimated at 0.51, reflecting an average reduction of 2.4 cm (week 0 vs. week 8; 91.72 ± 5.35 cm vs. 88.97 ± 5.36 cm). The analysis was conducted with a significance level (*α*) of 0.05, desired power of 0.80. The results indicated that a sample size of approximately 11 participants per group (a total of 33 participants across the three arms) would be required to achieve the desired power.

All statistical analysis of raw data was conducted using SPSS and Microsoft Excel. A *p*‐value of <0.05 was considered statistically significant for all tests. Normality was examined using Shapiro–Wilk test, with data presented as mean (SD) and median (IQR) for normally and not normally distributed data. One‐way analysis of variance (ANOVA) was utilized to compare measures between groups, independently at baseline and post‐intervention timepoints, of normally distributed data and Kruskal–Wallis test for not normally distributed data.

Primary analysis of the interaction between groups and time was conducted using one‐way analysis of covariance (ANCOVA) of absolute change scores with baseline values as covariates, with the Bonferroni test used for post‐hoc analysis. Secondary comparisons for within‐subjects data (baseline vs. post‐intervention) were performed using the Student paired *t*‐test for parametric variables or Wilcoxon signed‐rank test for non‐parametric variables.

## 3 RESULTS

### 3.1 Baseline characteristics

Table 1 displays the baseline characteristic of all study participants. There was a total of 5 dropouts. In the remaining total cohort (*n* = 30), participants mean age was 56.7 years. There were no differences in baseline characteristics among the three study arms (*p* > 0.05) except for weight (*F*_(2,29)_ = 4.03, *p* = 0.029) and hip circumference (HC) (*F*_(2,29)_ = 4.02, *p* = 0.03). The CTL group had significantly higher weight and HC scores than EX and EX+D groups (*p* < 0.05) (Table 1).

**TABLE 1:** Baseline physical characteristics of total cohort.

General characteristics	Total (*n* = 30)	CTL (*n* = 10)	EX only (*n* = 10)	EX+D (*n* = 10)	*p*
Age	56.7 ± 3.9	54.7 ± 3.3	57.7 ± 4.8	57.4 ± 3.3	0.25
Years of Menopause (years)	6.5 (3.0, 10.0)	4 (2, 7.5)	8.1 ± 3.3	6.5 (3, 11)	0.43
Weight (kg)	81.5 (68.5, 88.6)	93.9 ± 24.0	77.1 ± 9.8	75.7 ± 10.5	**0.029**
Height (m)	1.64 ± 0.06	1.67 ± 0.07	1.61 ± 0.05	1.64 ± 0.05	0.14
BMI (kg/m^2^)	28.8 (26.4, 32.7)	31.5 (27.6, 42.0)	29.7 ± 3.5	28.2 ± 3.4	0.05
Waist (cm)	92.8 ± 10.8	92.0 (76.3, 120.3)	91.9 ± 8.4	88.7 ± 9.4	0.16
Hip (cm)	114.1 ± 13.3	124.3 ± 16.4	109.2 ± 6.1	110.1 ± 9.2	**0.03**
Waist to hip ratio (WHR)	0.81 ± 0.06	0.80 ± 0.07	0.84 ± 0.06	0.81 ± 0.05	0.33
VAT (g)	1102.89 ± 531.1	1312.2 ± 769.3	1177.9 ± 383.1	768.5 ± 361.7	**0.016**

*Note*: Data presented as mean ± SD or median (IQR).

Abbreviations: BMI, body mass index; VAT, visceral adipose tissue.

### 3.2 Adherence and compliance to the interventions

#### 3.2.1 HEFHIT

Two participants from EX and one participant from EX+D dropped out during the intervention. Health status and family‐related reasons were the main factors to participation. No participants were injured during the intervention.

Ten participants each in EX and EX+D completed the intervention. The average adherence rate was 85% (21 out of 24 sessions) and 96% (23 out of 24 sessions) for EX and EX+D, respectively. Self‐reported HIIT intervals ranged from 20 s to 2 min for the first 4 weeks, and 10–60 s from weeks 5–8. Rest/low intervals ranged from 15 s to 4 mins for the first 4 weeks, and 10 s to 2 min during week 5–8.

Participants in both groups reached age‐related target HIIT zones (≥80% HRmax), with an average HR and average maximum HR of 96% and 122%, respectively for EX, and 98% and 124%, respectively for EX+D. The average rating for RPE scores were 18 for EX and 18 for EX+D (Table 2).

**TABLE 2:** Summary of adherence variables to the exercise intervention in both EX and EX+D groups.

	EX (*n* = 10)	EX+D (*n* = 10)	*p*
Completed Sessions *n* (%)	21 (85%)	23 (96%)	0.05
Average RPE	18.4 ± 0.4	18.0 ± 0.8	0.10
Average age‐related HR (%)	96.0 ± 6.6	98.1 ± 8.1	0.59
Average age‐related maximum HR (%)	122.1 ± 7.2	123.5 ± 6.5	0.69
Average calories expended/session	123.1 ± 34	124.9 ± 22.2	0.50

Abbreviations: HR, heart rate; RPE, rate of perceived exertion.

#### 3.2.2 Mediterranean diet

Mean adherence percentages across the 8 weeks showed 80% (*n* = 8) of participants in EX+D had optimal adherence. Of the ten participants, the baseline mean MEDAS scores and percentage were 6.6 ± 2.8 and 56.4 ± 16.9%, respectively. At post‐intervention, all participants improved their adherence to MD, with significant increases in the mean MEDAS score by 4 points (post: 10.6 ± 2.9) and percentage adherence of 23.2% (post: 79.6 ± 17.3; *p* = 0.002). Throughout the 8 weeks, 50% of participants (*n* = 5) maintained optimal adherence to MD (92.6 ± 0.1%). Of the remaining participants, 30% (*n* = 3) maintained optimal adherence (77.8 ± 0.1%) for 7 weeks, 10% (*n* = 1) for 2 weeks (71.4 ± 0.0), and the remaining participant did not adhere throughout the 8 weeks (56.8 ± 0.1%).

### 3.3 Intervention effect on body composition

There were no significant differences in baseline or main intervention effects for VAI (*p* > 0.05), between or within groups, respectively (Table 2).

However, there was a significant main effect for time for all anthropometric variables (Table 2).

Compared to CTL, the difference in weight change between CTL and EX over time is 1.8 ± 0.6 (SE) kg, (*p* < 0.05) and EX+D is 2.5 ± 0.6 (SE) kg, the difference between EX and EX+D would be 0.9 ± 0.5 (SE) kg (*p* = 0.25), estimated effect size is reported as *η* ^2^ = 0.423. BMI and WC (*p* < 0.01, *η* ^2^ = 0.417; *p* < 0.05, *η* ^2^ = 0.256, respectively) also significantly decreased by 1.5 ± 0.2 (SE) kg/m^2^ and 1.7 ± 0.8 (SE) cm in EX and 1.8 ± 0.2 (SE) kg/m^2^ and 1.4 ± 0.8 (SE) cm in EX+D, when compared to control. There were also significant differences for HC (*p* = 0.01, *η* ^2^ = 0.230) and body fat mass (*p* = 0.01, *η* ^2^ = 0.299) between CTL versus EX+D. EX group also demonstrated a significant reduction in VAT mass (*p* < 0.01, *η* ^2^ = 0.539) when compared to CTL. Conversely, there is a significant difference inlean mass when compared to CTL, indicating a decrease in lean mass when compared to control (*p* = 0.02, *η* ^2^ = 0.228).

Within group effects, indicates in EX+D, there were significant changes in all anthropometric variables in line with improvement (weight: −1.5 kg, *p* = 0.005; BMI: −0.6 kg/m^2^, *p* = 0.004; WC: −1.8 cm, *p* = 0.005; HC: −2.6 cm, *p* = 0.03; body fat mass: −2.4 kg, *p* = 0.004; lean mass: 0.9 kg, *p* = 0.049; VAT mass: −114.1 g, *p* = 0.01). In EX, there was a significant decrease in WC (−2.1 cm, *p* = 0.02) and VAT mass (− 327.5 g, *p* = 0.002). In CTL, there were significant increases in weight (1.2 kg, *p* = 0.003) and BMI (0.4 kg/m^2^, *p* = 0.009).

### 3.4 Intervention effects on leptin and adiponectin

There were no significant differences in baseline or main intervention effects for leptin (*p* > 0.05), adiponectin (*p* > 0.05), or adiponectin: leptin (ADI:LEP) (*p* = 0.05) between or within groups, respectively (Table 3).

**TABLE 3:** Baseline and post‐intervention adiposity markers.

Variable		Groups			*p* (G)
		CTL (*n* = 10)	EX (*n* = 10)	EX+D (*n* = 10)	
Weight (Kg)	Pre	93.9 ± 24.0	77.1 ± 9.8^#^	75.7 ± 10.5^#^	**<0.001**
	Post	95.0 ± 24.4	76.4 ± 10.2^##^	74.2 ± 9.9^###^	
	Δ	+1.2 ± 0.8	−0.6 ± 1.2	−1.5 ± 1.3	
	*p*	**0.003**	0.2	**0.005**	
BMI (Kg/m^2^)	Pre	33.5 ± 6.8	29.7 ± 3.5	28.2 ± 3.4	**<0.001**
	Post	33.9 ± 6.9	29.5 ± 3.6^##^	27.7 ± 3.1^###^	
	Δ	+1.2 ± 0.8	−0.3 ± 0.5	−0.6 ± 0.5	
	*p*	**0.009**	0.2	**0.004**	
Waist circumference (cm)	Pre	99.1 ± 12.7	91.9 ± 8.4	88.7 ± 9.4	**0.024**
	Post	98.7 ± 13.1	89.9 ± 9.6^##^	86.9 ± 9.8^#^	
	Δ	−0.4 ± 2.6	−2.1 ± 2.2	−1.8 ± 1.5	
	*p*	0.6	**0.02**	**0.005**	
Hip circumference (cm)	Pre	124.3 ± 16.4	109.2 ± 6.1	110.1 ± 9.2	**0.029**
	Post	122.9 ± 17.7	108.8 ± 7.8	107.5 ± 8.5^#^	
	Δ	0.4 ± 1.2	−0.5 ± 2.5	−2.6 ± 3.2	
	*p*	0.3	0.6	**0.03**	
Body fat (kg)	Pre	44.5 ± 15.3	34.1 ± 6.9	32.7 ± 7.3	**0.041**
	Post	44.6 ± 16.0	34.1 ± 7.3	29.7 ± 6.9	
	Δ	0.1 ± 1.5	0.0 ± 1.5	−2.4 ± 2.0	
	*p*	0.87	0.98	**0.004**	
Lean Tissue Mass (Kg)	Pre	49.7 ± 9.1	42.2 ± 4.5^#^	43.8 ± 5.0^#^	**0.035**
	Post	50.7 ± 8.9	42.5 ± 4.3^#^	44.5 ± 4.3	
	Δ	1.0 ± 1.5	−0.3 ± 1.5	0.9 ± 1.3	
	*p*	0.06	0.56	0.05	
VAT (g)	Pre	1312.2 ± 769.3	1177.9 ± 383.1	768.5 ± 361.7	**0.016**
	Post	1388.8 ± 802.7	850.4 ± 376.2^##^	654.4 ± 375.0	
	Δ	−76.7 ± 70.2	−327.5 ± 233.8	−114.1 ± 74.2	
	*p*	0.28	**0.002**	**0.01**	
Visceral Adiposity Index	Pre	2.2 ± 0.9	1.9 ± 0.8	1.6 ± 0.7	0.63
	Post	2.0 ± 1.1	1.9 ± 0.7	1.5 ± 0.6	
	Δ	−0.2 ± 0.7	0.0 ± 0.6	−0.1 ± 0.6	
	*p*	0.44	0.43	0.46	
Leptin ng/mL	Pre	61.1 ± 31.3	43.5 ± 14.1	24.1 (18.6, 37.8)	0.44
	Post	57.3 ± 22.2	26.6 (23.0, 57.6)	20.1 (13.2, 40.7)	
	Δ	9.2 ± 7.2	−1.4 ± 27.4	−2.8 ± 10.5	
	*p*	0.32	0.51	0.29	
Adiponectin ng/mL	Pre	6.8 ± 2.0	5.4 ± 2.9	5.8 (4.7, 21.6)	0.37
	Post	5.9 ± 2.3	5.7 (4.4, 10.8)	5.7 ± 1.8	
	Δ	−0.9 ± 2.9	−0.1 (−0.7, 2.0)	0.7 (−16.0, 1.6)	
	*p*	0.23	0.27	0.27	

*Note*: Data are presented as mean ± SD or median (IQR) for normally and not normally distributed data, respectively. # Denotes significance at independent timepoint when compared to CTL. G: Between group effect, applies to absolute change score only. P: Within‐group effect, applies to absolute change score only. Statistically significant value (*p* < 0.05) highlighted in bold.

Abbreviations: BMI, body mass index; VAT, visceral adipose tissue.

^#^ *p* < 0.05.

^##^ *p* < 0.01.

^###^ *p* < 0.001.

## 4 DISCUSSION

For the first time, we show that low‐cost, home‐based equipment‐free interval training with or without adoption of a Mediterranean diet significantly improved adiposity markers in postmenopausal women. Specifically, body composition (body weight, BMI, WC, HC, and VAT) improved in combined exercise and diet by the end of 8 weeks. Furthermore, compared to a control arm, body weight, BMI, and WC decreased in both exercise‐only and exercise‐and‐diet arms.

Previous meta‐analyses have confirmed the efficacy of combined exercise and dietary interventions on WC in obese postmenopausal women (Cheng et al., 2018; Khalafi & Symonds, 2023), supporting the findings in this study. Both intervention groups showed significant decreases in WC and VAT, highlighting the feasibility of both lifestyle behaviors in improving central adiposity (Khodadadi et al., 2023). Previously published literature investigating HIIT interventions predominantly utilize treadmills and cycle ergometers (Batacan et al., 2017), which may be unreflective of real‐world applications and may exclude individuals who lack accessibility to exercise facilities. Findings support high‐intensity interval exercise (HIIE) in improving post‐exercise energy homeostasis and metabolic adaptations, potentially due to its ability to increase post‐exercise fat oxidation and thereby mitigating body fat deposition (Atakan et al., 2022). In accordance with previous meta‐analyses, performing HIIT for at least 4 weeks have demonstrated efficacy in reducing abdominal adiposity (Andreato et al., 2019; Guo et al., 2023), supporting our findings. In addition to reductions in abdominal obesity, the supplementation of the MD to HEFIT elicited favorable weight loss compared to HEFIT alone, although this comparison was not significant. In agreement, meta‐analyses investigating the integration of diet and exercise have consistently demonstrated more favorable cardiometabolic health outcomes compared to diet or exercise alone in overweight post‐menopausal women (Cheng et al., 2018; Khalafi & Symonds, 2023). Both Cheng et al. (2018) and Khalafi & Symonds (2023) reported greater weight loss with combined exercise and diet compared to standalone interventions. Furthermore, the UK Women's Cohort Study linked long‐term adherence to the MD with improvements in WC and reduced risk of abdominal obesity in post‐menopausal women (Best & Flannery, 2023). In addition to favorable effects on body weight with combined interventions, this study highlights that HEFIT with or without MD can improve abdominal obesity in predisposed women, an important factor in mitigating cardiometabolic dysfunction.

No changes in leptin or adiponectin were observed across all groups. Although existing literature links alterations in leptin and adiponectin with exercise and/or diet‐induced weight loss, this association was not evident in our study despite significant weight loss observed in the EX+D group. The discrepancy may stem from the insufficient weight loss to elicit substantial changes in leptin and adiponectin. A meta‐analysis by Khalafi et al. found a decrease of 6 kg body weight that was associated with leptin and adiponectin change with combined exercise and diet (Khalafi et al., 2023). In a similar cohort to this study, a 3‐month weight loss program (hypocaloric diet and exercise) (Wooten et al., 2022) and 6‐month hypocaloric diet (Van Rossum et al., 2000) resulted in significant weight loss that also strongly associated with leptin and adiponectin change. This suggests that weight loss irrespective of method employed, is the primary determinant for changes in these adipokines.

Although investigating into the effects of continuous physical inactivity and habitual diet were not an objective in this study, the CTL group showed significant increases in body weight and BMI, signifying early indication of declines in cardiometabolic health. While the weight gain and increase in BMI cannot be explained due to unchanged body composition or nutritional intake, it should be noted that short‐term weight gain is associated with significantly increased odds of multi‐morbidity in middle‐aged women (Xu et al., 2019). Without targeted lifestyle modifications, individuals in the control group may have continued to engage in behaviors associated with metabolic dysfunction, such as unhealthy eating habits, sedentary lifestyle, and inadequate physical activity, further exacerbating their cardiometabolic risk profile over time. The comparisons of the intervention groups with CTL underscores the protective effects of incorporating either easily implementable lifestyle interventions in adiposity markers with the mitigation of cardiometabolic health decline.

This study is not without limitations. Firstly, a larger sample size would have allowed a better insight into the response to both exercise and diet, given the interindividual variation in compensatory responses with lifestyle interventions such as exercise (Mansfeldt & Magkos, 2023). Secondly, the CTL group exhibited significantly higher baseline weight and HC than the intervention group, this is a result of two participants having higher values, and this being amplified due to a small sample size. Finally, the incorporation of MEDIET scoring sheets into CTL and EX only, and the standardization of olive oil type (particularly with extra virgin olive oil (Garcia‐Serrano et al., 2021)) should be incorporated in future studies. Let alone participants self‐reported not to adhere to a MD upon study initiation and no MD guidance given or instructed, it is with caution that results should be interpreted as there is no specific scoring data assessed in comparative groups.

One of the strengths of this study was the adherence and compliancy to the prescribed interventions in both groups, with <20% drop‐out rate in either group. Poor adherence is known to be one of the pitfalls of exercise and dietary interventions that can hinder favorable outcomes. Lack of time and competing priorities are the main barriers to adherence to lifestyle behavioral modifications (Deslippe et al., 2023). This study investigated HIIT and MD in an unsupervised, free‐living environment with the aim to mitigate these barriers. Not only do findings from this study highlight the feasibility of HEFIT with/without MD in an unsupervised manner, but the potential cardiometabolic benefits of both behaviors if sustained for longer periods.

## 5 CONCLUSION

This study demonstrates the efficacy of regular exercise via feasible home‐based, equipment‐free interval training with or without the Mediterranean‐style diet in improving adiposity biomarkers such as waist circumference and visceral adipose tissue in overweight/obese postmenopausal women. Adding a Mediterranean diet to exercise training further enhanced cardiometabolic health, evidenced by clinically relevant weight loss. Such improvements, if sustained, might help maintain cardiometabolic health, suggesting that continued adherence can lead to significant long‐term benefits.

## AUTHOR CONTRIBUTIONS

A.T and R.C contributed to the experimental study design, conducted the pre‐ and post‐intervention assessments, assays, analysis and interpretation of data analysis and the drafting of the manuscript. G.D contributed to the assays, analysis and the drafting of the manuscript. S.L.P and R.M.B contributed to the experimental study design and the drafting of the manuscript. All authors (A.T, G.D, R.L.T, S.L.P, R.M.B, and R.C) read and approved on the final version of the manuscript.

## Supporting information


Table S1.

## Data Availability

The data that support the findings of this study are available from the corresponding author upon reasonable request.
