# Supplementary material for: Effect of home‐based exercise with or without a Mediterranean‐style diet on adiposity markers in postmenopausal women: A randomized‐control trial
Source: Physiol Rep. 2025 Feb 7;13(3):e70239. doi: 10.14814/phy2.70239 (PMC11805804; doi:10.14814/phy2.70239)
Supplement: Supplementary file 1 — Table S1. [file PHY2-13-e70239-s001.docx]

**Title**

Effect of home-based exercise with or without a Mediterranean style diet on adiposity markers in post-menopausal women: a randomised-control trial

**Authors**

Abbigail Tan^a^, Gareth Dunseath^b^, Rebecca L. Thomas^b^, Sarah L. Prior^b^, Richard M. Bracken^a^, Rachel Churm^a^

^a^ Applied Sports Technology, Exercise and Medicine (A-STEM) Research Centre, Faculty of Science and Engineering, Swansea University, Swansea, UK.

^b^ Diabetes Research Group, Grove Building, Swansea University, Swansea, UK

^c^ School of Nursing and Health Sciences, Sciences Complex, University of Sunderland, Sunderland, UK

^d^ Leeds Institute of Cardiovascular and Metabolic Medicine, University of Leeds, Leeds, UK

***Corresponding Author**

Rachel Churm

Applied Sports Technology, Exercise and Medicine (A-STEM) Research Centre

Faculty of Science and Engineering

Swansea University

Swansea

United Kingdom

Email: r.churm@swansea.ac.uk

Supplementary Material Table S1. Nutrient values from MyFitnessPal in all groups.

|  | | **CTL (n = 10)** | **EX only (n = 10)** | **EX+D (n = 10)** | ***P*** |
| --- | --- | --- | --- | --- | --- |
| **Total Energy (kcal/day)** | **Pre** | 1408.4 ± 217.4 | 1230 (1150.2, 1402.4) | 1527.3 ± 348.1 | 0.2 |
|  | **Post** | 1251.4 ± 132.6 | 1402.3 ± 351.7 | 1391.3 ± 236.1 |  |
|  | **Δ** | -157.0 ± 182.2 | 88.7 ± 163.1 | -136.1 ± 300.4 |  |
|  | ***P*** | 0.09 | 0.12 | 0.19 |  |
| **Carbohydrate (g)** | **Pre** | 148.0 ± 34.0 | 144.8 ± 29.0 | 156.1 (116.5, 173.3) | 0.22 |
|  | **Post** | 123.7 ± 10.9 | 129.7 ± 24.5 | 152.8 ± 46.2 |  |
|  | **Δ** | -24.2 ± 35.8 | -15.1 ± 30.5 | -5.3 ± 25.4 |  |
|  | ***P*** | 0.16 | 0.21 | 0.52 |  |
| **Fat (g)** | **Pre** | 53.5 ± 13.0 | 46.1 ± 22.0 | 62.9 ± 25.7 | 0.9 |
|  | **Post** | 49.2 ± 7.6 | 51.8 ± 18.3 | 53.6 ± 20.0 |  |
|  | **Δ** | -4.3 ± 12.1 | 5.7 ± 14.2 | -9.3 ± 33.1 |  |
|  | ***P*** | 0.43 | 0.29 | 0.39 |  |
| **Protein (g)** | **Pre** | 57.2 ± 15.0 | 51.8 ± 13.8 | 56.9 ± 15.0 | 0.25 |
|  | **Post** | 50.4 ± 14.2 | 58.1 ± 17.4 | 56.0 ± 16.1 |  |
|  | **Δ** | -6.7 ± 9.2 | 6.3 ± 12.6 | -0.9 ± 4.4 |  |
|  | ***P*** | 0.13 | 0.2 | 0.85 |  |
|  | **Post** | 2301.5 ± 572.8 | 1920.1(1833.0, 2288.0) | 1996.1 ± 190.1 |  |
|  | **Δ** | 27.2 (-6.0, 45.3) | 15.1 ± 278.4 | -37.0 ± 182.2 |  |
|  | ***P*** | 0.86 | 0.86 | 0.54 |  |

Supplementary Material Table S2. Validated 14-item Mediterranean diet adherence score (MEDAS) tool adapted from the PREDIMED study [14].

|  | Yes | No |
| --- | --- | --- |
| 1. Is olive oil the main culinary fat used? |  |  |
| 1. Are ≥ 4 tablespoons of olive oil used each day? |  |  |
| 1. Are ≥ 2 servings (of 200g each) of vegetables eaten each day? |  |  |
| 1. Are ≥ 3 servings of fruit (of 80g each) eaten each day? |  |  |
| 1. Is < 1 serving (100-150g) of red meat/ hamburgers/ other meat products eaten each day? |  |  |
| 1. Is < 1 serving (12g) of butter, margarine or cream eaten each day? |  |  |
| 1. Is < 1 serving (330ml) of sweet or sugar sweetened carbonated beverages consumed each day? |  |  |
| 1. Are ≥ 3 glasses (of 125ml) of wine consumed each week? |  |  |
| 1. Are ≥ 3 servings (of 150g) of legumes consumed each week? |  |  |
| 1. Are ≥ 3 servings of fish (100-150g) or seafood (200g) eaten each week? |  |  |
| 1. Is < 3 servings of commercial sweets/pastries eaten each week? |  |  |
| 1. Is ≥ 1 serving (of 30g) of nuts consumed each week? |  |  |
| 1. Is chicken, turkey or rabbit routinely eaten instead of veal, pork, hamburger or sausage? |  |  |
| 1. Are pasta, vegetable or rice dishes flavoured with garlic, tomato, leek or onion eaten ≥ twice a week? |  |  |
| **TOTAL SCORE** (total number of ‘yes’ answers) |  | |
